# Effects of cold acclimation on serum biochemical parameters and metabolite profiles in *Schizothorax prenanti*

**DOI:** 10.1186/s12864-024-10483-z

**Published:** 2024-06-01

**Authors:** Aiyu Wang, Run Zhang, Xianshu Zhang, Chunjie Chen, Quan Gong, Linjie Wang, Yan Wang

**Affiliations:** 1 College of Animal Science and Technology, Sichuan Agricultural University, Chengdu, Sichuan 611130 China; 2 Fisheries Institute, Sichuan Academy of Agricultural Sciences, Chengdu, Sichuan 611713 P.R. China

**Keywords:** Cold acclimation, Metabolomics, Amino acids, Unsaturated fatty acids, *Schizothorax prenanti*

## Abstract

**Background:**

Environmental temperature is critical in regulating biological functions in fish. *S. prenanti* is a kind of cold-water fish, but of which we have little knowledge about the metabolic adaptation and physiological responses to long-term cold acclimation.

**Results:**

In this study, we determined the physiological responses of *S. prenanti* serum after 30 days of exposure to 6℃. Compared with the control group, the levels of TC, TG, and LDL-C in the serum were significantly (*P* < 0.05) increased, and the level of glucose was significantly (*P* < 0.05) decreased under cold acclimation. Cold acclimation had no effect on the gene expression of pro-inflammatory factors and anti-inflammatory factors of *S. prenanti*. Metabolomics analysis by LC-MS showed that a total of 60 differential expressed metabolites were identified after cold acclimation, which involved in biosynthesis of amino acids, biosynthesis of unsaturated fatty acids, steroid degradation, purine metabolism, and citrate cycle pathways.

**Conclusion:**

The results indicate that cold acclimation can alter serum metabolites and metabolic pathways to alter energy metabolism and provide insights for the physiological regulation of cold-water fish in response to cold acclimation.

**Supplementary Information:**

The online version contains supplementary material available at 10.1186/s12864-024-10483-z.

## Introduction

 Environmental temperature is an important abiotic factor affecting aquatic organisms [1, 2]. As an aquatic poikilothermy, fish have no ability to regulate their body temperature, which will change with the external environment [3]. Due to the increasing frequency and intensity of extreme weather events caused by global climate change, variations of ambient temperature have attracted strong attention from aquaculture and fisheries researchers [4]. Water temperature rise caused by climate change damages the growth performance [5], disrupts embryonic development [6], causes oxidative damage to the fish [7], and leads to a wastage in the quality of fish fillets [8]. Additionally, extreme temperature alters the spawning season and feeding grounds of fish, as well as the abundance of dominant species [9]. More seriously, the cold snaps caused by climate change can lead to mass mortalities of fish [10, 11]. Therefore, how to alter and regulate the physiological responses of fish to cope with ambient temperature changes is an important issue.

It is reported that among tropical fish, discus fish is one of the most popular species in the warm water aquarium, inhabiting at a temperature of 24–33℃. Under the low temperature of 20℃, discus fish changes its metabolites and activates glutathione metabolism to adapt to the cold [12]. In addition, tropical teleost tilapia exposed to 15 °C lowers oxygen consumption rate by about 75.11% [13]. *Astyanax lacustris*, as a subtropical freshwater fish, shows high energy demand within 48 h of exposure to 15℃, which is involved in glycolysis, citric acid cycle, and amino acid catabolism pathways [14]. Obviously, fish may adapt to the environment by regulating their metabolism to reduce energy demand and consumption when facing low temperature. The optimal growth temperature of farmed tilapia is 22–35℃. Under the low-temperature stress condition of (9 ± 1)℃, the serum C3, C4, and lysozyme immune indicators significantly decrease in farmed tilapia, indicating that low temperature affects the non-specific immunity of farmed tilapia [15]. It can be seen that tropical fish may alter their metabolism and immune status at low temperature.

Cold-water fish may have different behaviors and physiological reactions at low temperatures. In cold-water fish, the optimal growth water temperature range of rainbow trout is 7.8–19.2℃ [16]. According to reports, cold stress significantly increases the activity of serum alanine transaminase in rainbow trout [17]. In addition, cold stress affects the lipid metabolism of dorsal muscle tissue in *Phoxinus lagowskii* through an increase in polyunsaturated fatty acids and changes in glycolipid metabolic enzymes [18]. However, cold stress does not affect plasma lactate, ion concentrations, and osmotic pressure in *Salmo salar* [19], indicating that cold stress has few physiological effects on *Salmo salar*. Interestingly, it is reported that fish living in polar regions with low water temperature throughout the year have evolved physiological mechanisms to adapt to cold, including the synthesis of antifreeze protein (AFP), antifreeze glycoprotein (AFGP), and molecular chaperones (heat shock proteins) in plasma [20, 21].

The cold-water fish may undergo a series of physiological reactions to counteract the passive effects of temperature reduction, and different species may have different physiological responses to low temperatures. In addition, the cold response of bony fish to temperature can be classified as acute stress and chronic acclimation. Recently, researchers have conducted more research on the effects of short-term acute cold stress on fish. However, the metabolic adaptation mechanism and physiological responses of serum in cold-water fish to long-term cold acclimation are still unknown.

*Schizothorax prenanti* (*S. prenanti*) belongs to the Schizothoracinae subfamily of the Cyprinidae family. *S. prenanti* mainly is distributed in the upper reaches of the Yangtze River, and known as the Ya-fish in Sichuan Province. *S. prenanti* can grow in the temperature range of 5–27℃, and the optimal growth temperature is 21℃ [22], which belongs to a type of cold-water fish. At present, *S. prenanti* is an important valuable economic fish and widely cultivated in Southwestern China [23]. However, we have little knowledge about the metabolic adaptation and physiological responses of *S. prenanti* under cold acclimation. In this study, we adopted a battery of serum biochemical indicators combined with LC-MS metabolomic analysis to determine the metabolites involved in the process of cold acclimation. In addition, we used the expression of pro-inflammatory and anti-inflammatory factors to determine the effects of cold acclimation on the immune status of *S. prenanti*. This study will help to understand the potential mechanisms by which cold-water fish adapt to low temperature.

## Result

### Cold acclimation has no effects on growth performance but changes biochemical indicators in* S. prenanti*

To determine the effects of cold acclimation on the growth performance of *S. prenanti*, we measured the initial and final weights from 30 d to 75 d. As shown in Table 1, there was no difference in weight gain rate (WGR), specific growth rate (SGR), and feed conversion rate (FCR) between the cold and control groups, indicating that the growth performance of *S. prenanti* is not be affected under cold acclimation.

**Table 1:** Growth performance of *S. prenanti* in the control and cold groups (*n* = 15, fish per group)

Groups	Initial weight (g)	Final weight (g)	WGR (%)	SGR (%)	FCR
Control	125.00 ± 2.67	131.93 ± 3.27	5.51 ± 0.86	0.08 ± 0.01	2.24 ± 0.10
Cold	124.87 ± 3.44	130.57 ± 3.48	4.58 ± 0.21	0.06 ± 0.01	2.43 ± 0.04

Data are presented as mean ± SEM (standard error of mean)

The calculation formula is as follows: weight gain ratio (WGR, %) = (Wt-W0)/W0 × 100%;

$$\mathrm{specific}\;\mathrm{growth}\;\mathrm{rate}\;(\mathrm{SGR},\;\%)\;=\;(\mathrm{lnWt}-\mathrm{lnW}0)/\mathrm t\times100\%;\;$$

$$\mathrm{feed}\;\mathrm{conversion}\;\mathrm{rate}\;(\mathrm{FCR})\;=\;\mathrm F/(\mathrm{Wt}-\mathrm W0).\;\mathrm{In}\;\mathrm{the}\;\mathrm{formula},$$

W_t_: the final weight of the fish at the end of the experiment;

W_0_: the initial weight of the fish at the beginning of the experiment;

t: the time of the experiment;

F: feed intake.

**Fig. 1:**
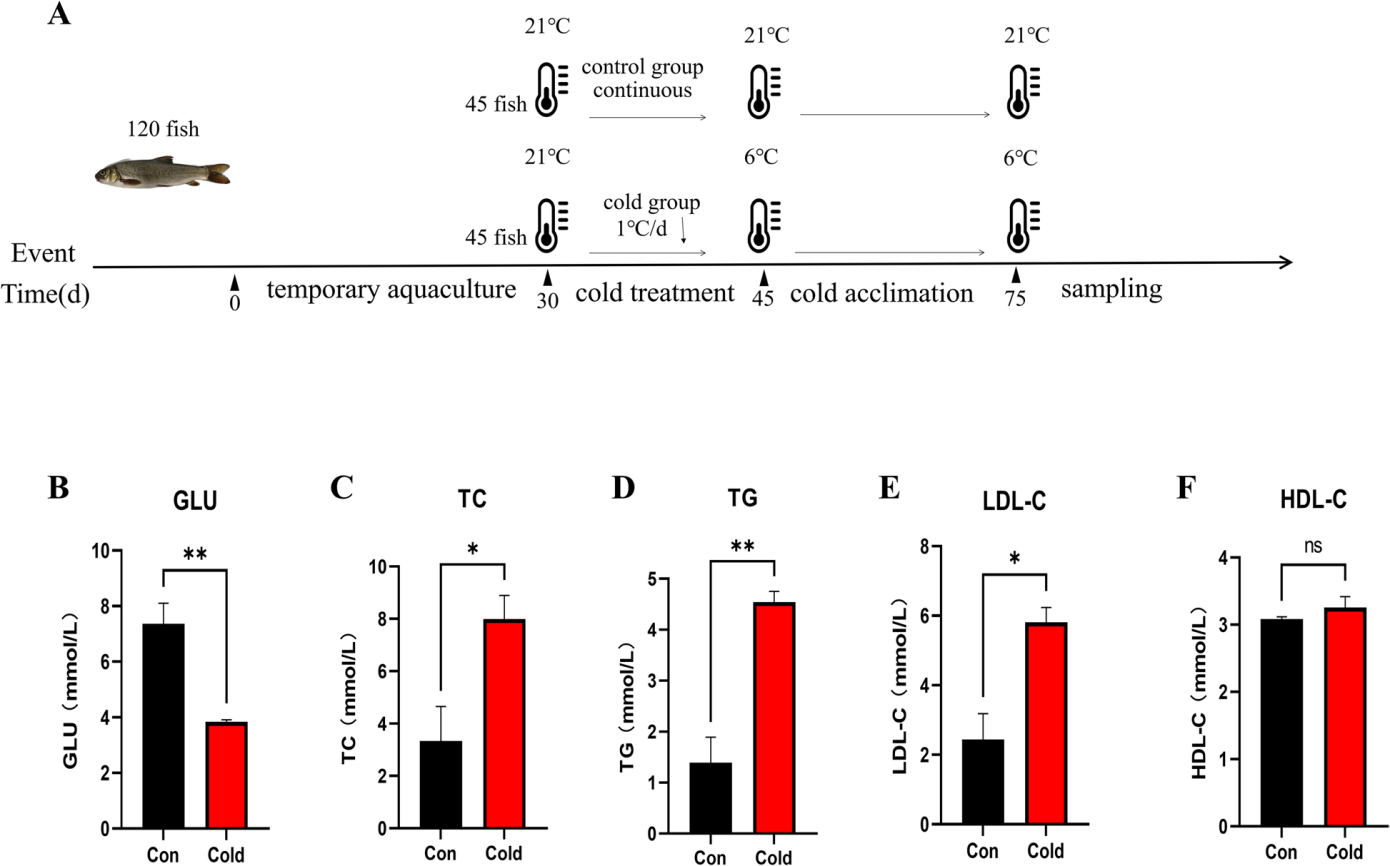
Experimental design diagram (**A**) and Serum biochemical parameters of *S. prenanti* exposed to control (black) and cold (red) groups. The indicators include GLU (**B**), TC (**C**), TG (**D**), LDL-C (**E**), and HDL-C (**F**). Data are shown as means ± SEM. *n* = 8, * and ** represent *P*  < 0.05 and *P*  < 0.01, respectively. Ns represents no significant difference between groups

To determine the effects of cold acclimation on the physiological responses of *S. prenanti*, we conducted biochemical assays on the serum. Compared with the control group, the levels of TG, TC, and LDL-C were significantly (*P* < 0.05) increased in the serum of the cold group. However, the level of glucose (GLU) was significantly (*P* < 0.05) decreased in the serum of the cold group. In addition, the level of HDL-C showed no difference compared to the control group (*P* > 0.05) (Fig. 1B-F). These results indicate that cold acclimation can change the levels of serum biochemical indicators of *S. prenanti*.

### Cold acclimation has no effects on the immune status of * S. prenanti*

**Fig. 2:**
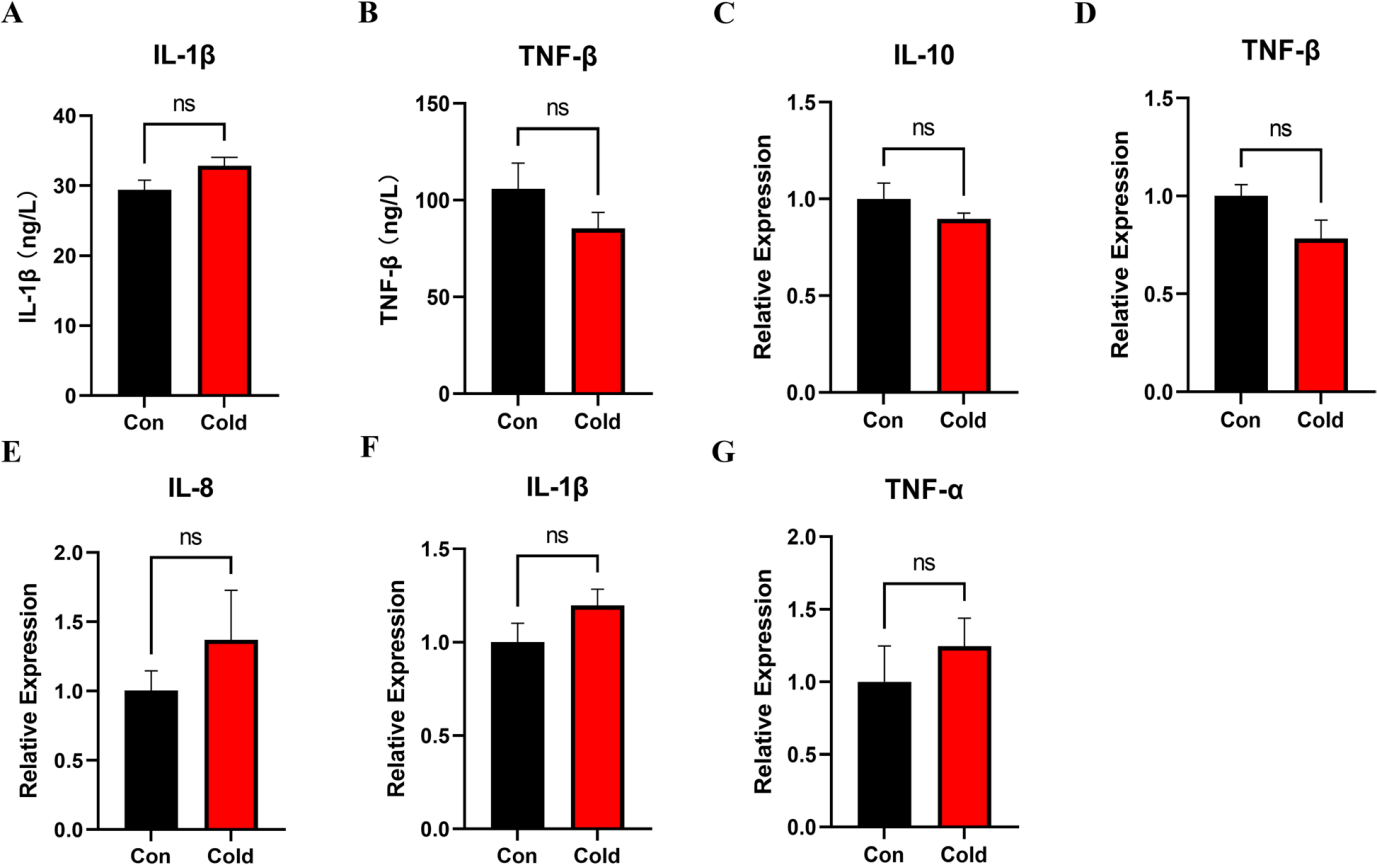
Serum ELISA and the expression levels of inflammation related genes in intestine of *S. prenanti* under cold acclimation. Serum ELISA includes IL-1β (**A**) and TNF-β (**B**) (*n* = 8). The expression levels of genes include anti-inflammatory factors IL-10 (**C**) and TNF-β (**D**) and pro-inflammatory factors IL-8 (**E**), IL-1β (F) and TNF-α (**G**) (*n* = 7). Data are shown as means ± SEM, ns represents no significant difference between treatments (*P >* 0.05)

Next, we examined the expression of pro-inflammatory and anti-inflammatory factors to investigate the effect of cold acclimation on the immune status of *S. prenanti*. Firstly, as typical pro and anti-inflammatory factors, serum IL-1β and TNF-β levels were determined by ELISA (Fig. 2A-B). We found that pro-inflammatory factor IL-1β and anti-inflammatory factor TNF-β were not significantly changed (*P* > 0.05) under cold acclimation. Subsequently, to expand our knowledge to other factors, the intestinal gene expression levels in other cytokines were examined (Fig. 2C-G). We found that there was no significant difference in the anti-inflammatory factors IL-10 and TNF-β between cold and control groups (*P* > 0.05), and there was no significant difference in the pro-inflammatory factors IL-1β, IL-8, TNF-α as well. The above results indicate that cold acclimation has no effects on the immune status of *S. prenanti* and may not cause intestine inflammation in *S. prenanti*.

### Cold acclimation changes the overall serum metabolites

**Fig. 3:**
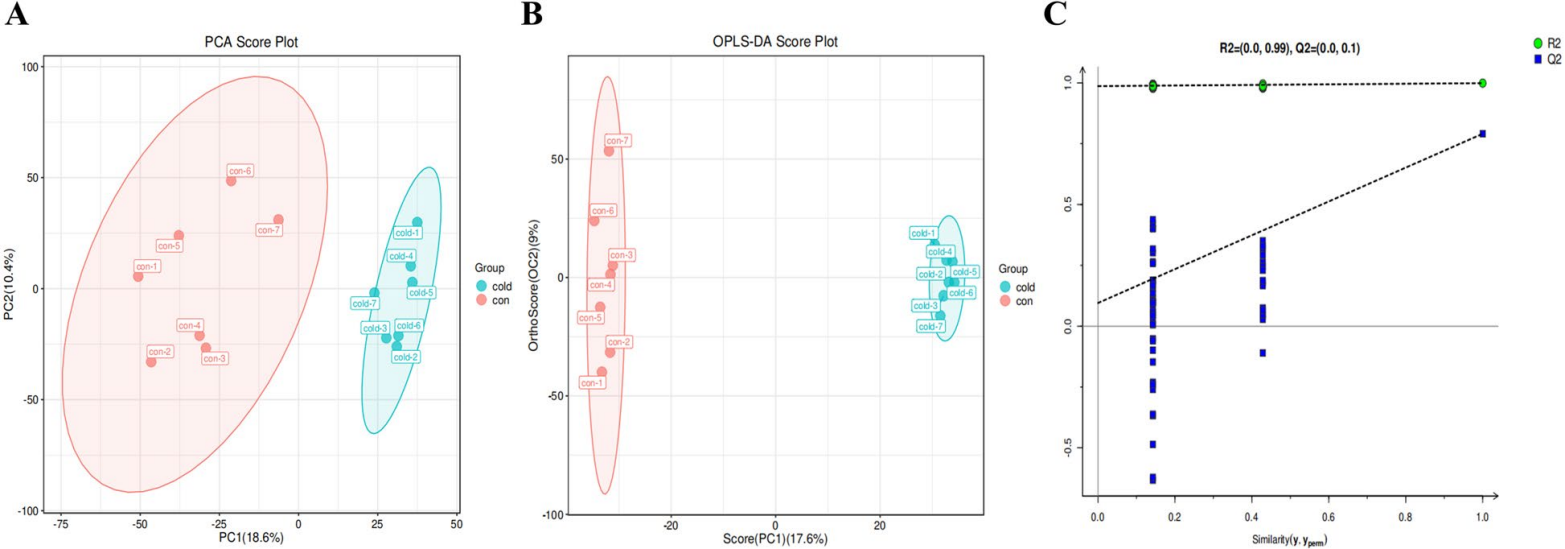
The diagram of overall changes in metabolites under cold acclimation. **A** PCA score plot of the serum samples from the two groups (*n* = 7); **B** OPLS-DA for discriminating the metabolite profiles of serum of the control (red) and cold (blue) groups, with each dot representing one serum sample from each treatment (A and B); **C** OPLS-DA permutation test chart. All blue Q2 points are lower than the rightmost original blue Q2, indicating that the structure is reliable and effective

To determine the overall changes in metabolites under cold acclimation, we extracted serum for metabolite detection. A total of 404 metabolites were detected by LC-MS (Table S2). PCA analysis was used to show a significant separation of the control and cold groups (Fig. 3A), indicating changes in metabolic spectra after cold treatment. We further employed OPLS-DA to identify differences in metabolite levels between the control and cold groups. The scatter plot of LC-MS data in the training set showed a significant difference between the two experimental groups (R2X = 0.267, R2Y = 0.999, Q2 = 0.791) (Fig. 3B). And the OPLS-DA permutation test chart (Fig. 3C) showed that the results are reliable. These results demonstrate that there is an obvious separation in metabolic changes between control and cold groups.

### Analysis of differential metabolites in serum under cold acclimation

**Fig. 4:**
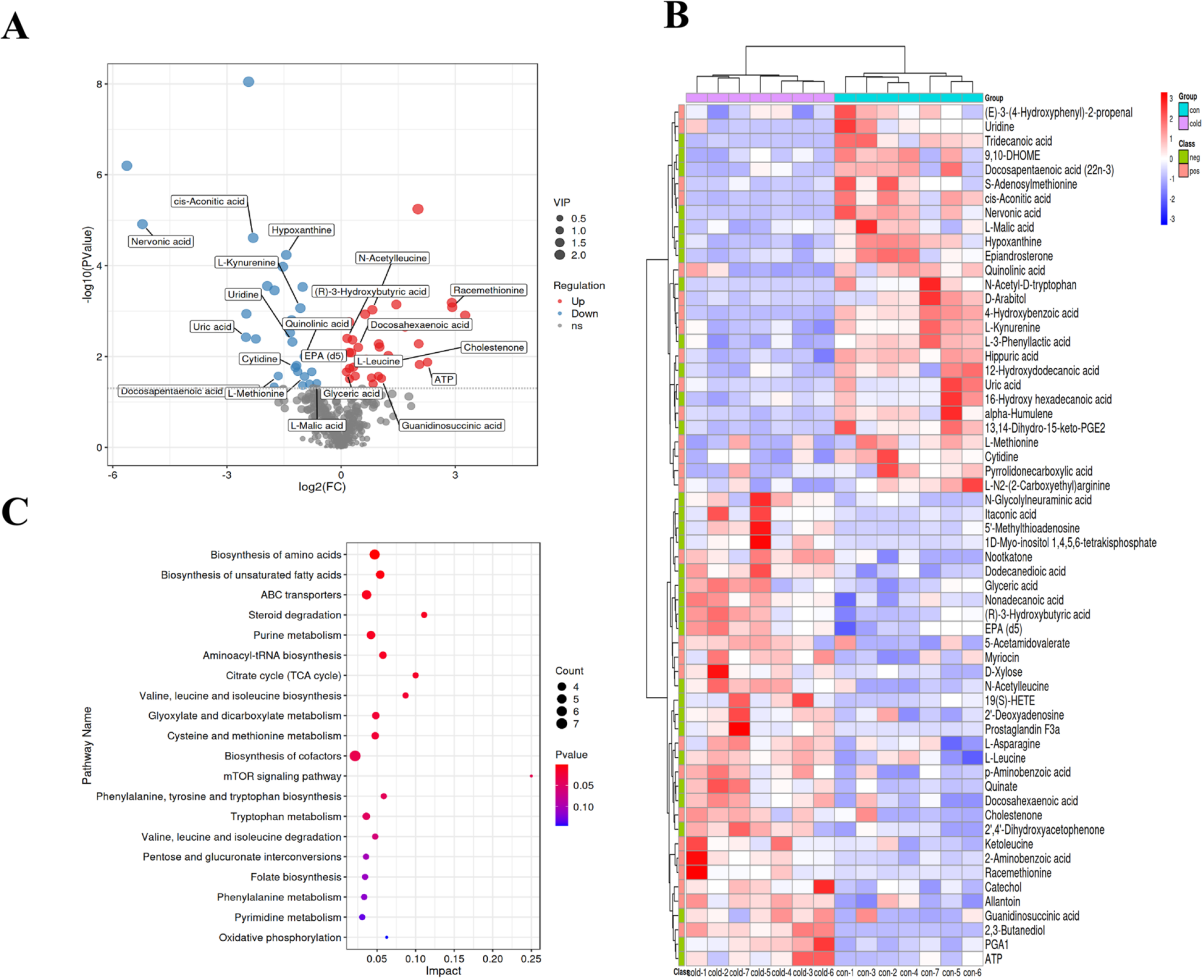
Identified differential expressed metabolites and metabolic pathway diagram. **A** Differential metabolite volcano plot between control group and cold group. Each dot in the plot represents a metabolite. **B** Heat-map visualization of differential metabolites in response to different groups. **C** Bubble plot of metabolic pathway influencing factors

A total of 60 differential expressed metabolites were screened with significant differences among 404 metabolites, which were shown in the volcanic plot (Fig. 4A), including 33 up-regulated and 27 down-regulated metabolites. Furthermore, we plotted a heat map of differential metabolites based on their relative quantity (Fig. 4B). The pathway analysis results provided a detailed account of the changes in the top 20 metabolic pathway changes associated with cold acclimation (Fig. 4C).

**Fig. 5:**
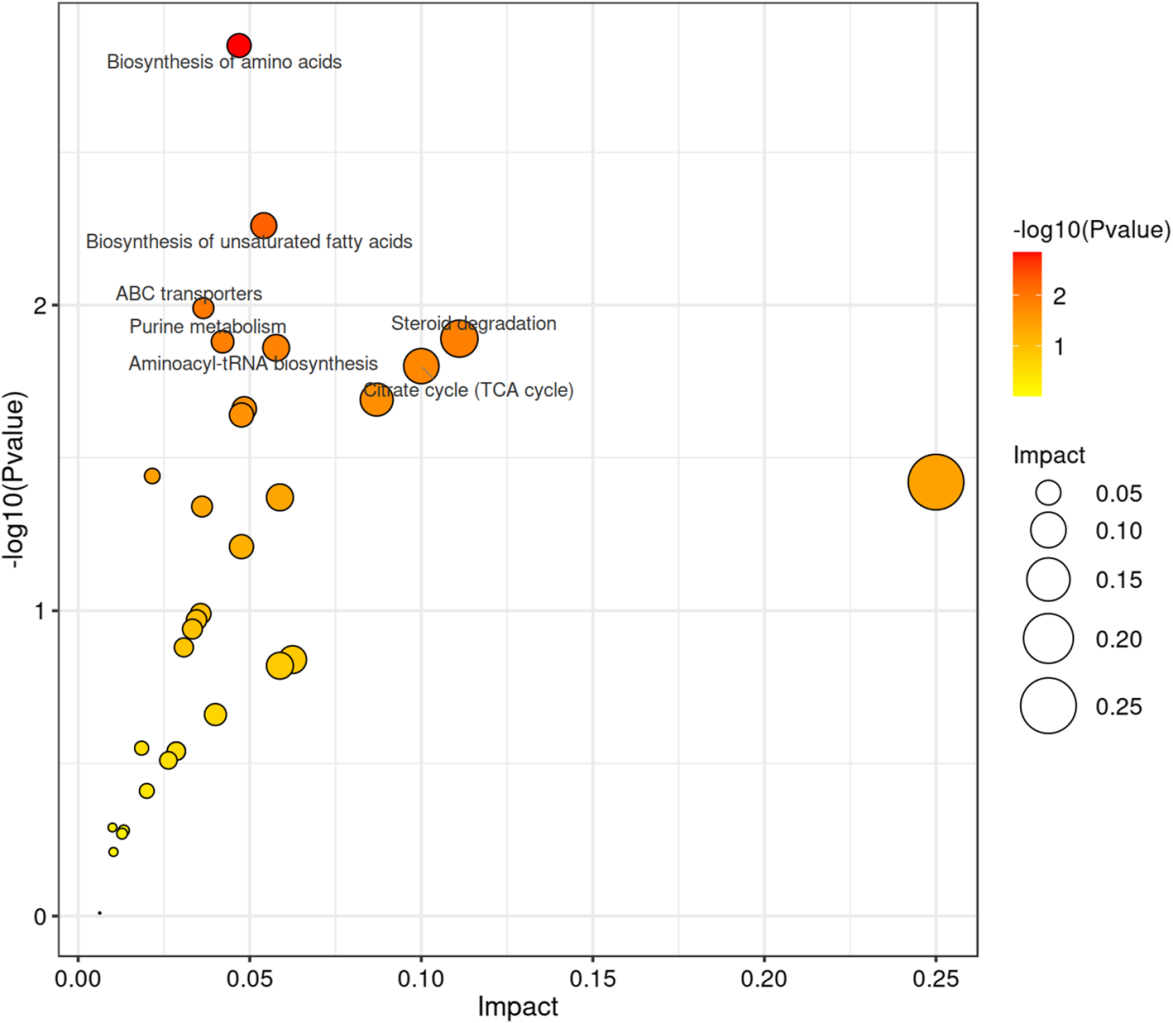
The top 7 pathway mapping based on differential expressed metabolites

The pathways of top 7 included biosynthesis of amino acids, biosynthesis of unsaturated fatty acids, ABC transporters, steroid degradation, purine metabolism, aminoacyl-tRNA biosynthesis, and citrate cycle (TCA cycle) (Fig. 5).

### Amino acid metabolism is induced in response to cold acclimation

To investigate the effects of cold acclimation on amino acid metabolism in *S. prenanti*, we found that the biosynthesis of amino acids was significantly (*P* < 0.05) enriched through the KEGG enrichment pathway under cold acclimation (Fig. 4C). Further analysis found that the levels of L-leucine, L-asparagine, and Ketolecine were significantly (*P* < 0.05) increased under cold acclimation, while the levels of S-adenosylmethionine and L-methionine were significantly (*P* < 0.05) decreased (Table 2). These results indicate that cold acclimation induces the biosynthesis of amino acids pathway in *S. prenanti*.

**Table 2:** Differential expression of amino acids under cold acclimation (control vs. cold)

Metabolites name	Fold change	*P* - value^a^	VIP value^b^	Trend^c^
S-adenosylmethionine	0.21	0.00410	1.68118	down*
L-methionine	0.52	0.02745	1.40186	down*
L-leucine	1.16	0.01829	1.51087	up*
L-asparagine	1.29	0.02684	1.43965	up*
Ketoleucine	1.74	0.02935	1.37085	up*

*Represents significantly changed metabolites (*P* < 0.05 and VIP > 1 were set as the threshold for significantly changed metabolites)

^a^*P* - value was calculated from Student's *t*-test

^b^VIP value, variable influence on projection (VIP) value was obtained from the OPLS-DA

^c^Up and down indicate an upward and downward trend in metabolites under cold acclimation

### Fatty acids metabolism is induced under cold acclimation

Interestingly, we found that the biosynthesis of unsaturated fatty acids was significantly (*P* < 0.05) enriched under cold acclimation (Fig. 4C). Further analysis indicated that the levels of eicosapentaenoic acid (EPA) and docosahexenoic acid (DHA) were significantly (*P* < 0.05) increased in the cold group. However, the level of docosapentaenoic acid (DPA) was significantly (*P* < 0.05) decreased (Table 3). These results indicate that the content of polyunsaturated fatty acids in *S. prenanti* serum is increased under cold acclimation.

**Table 3:** Differential expression of unsaturated fatty acid in serum of *S. prenanti* under cold acclimation (control vs. cold)

Metabolites name	Fold change	*P* - value^a^	VIP value^b^	Trend^c^
Palmitoleic acid (C16:1)	1.19	0.48178	0.44737	up
Octadecenoic acid (18:1)	0.45	0.09230	1.05620	down
Oleic acid (C18:1)	2.06	0.25598	0.72284	up
Linoleic acid (C18:2)	0.49	0.14345	1.01444	down
Alpha-Linolenic acid (C18:3)	0.9	0.23952	0.69433	down
Gamma-Linolenic acid (C18:3)	0.8	0.32881	0.62678	down
Arachidonic acid (C20:4)	0.97	0.89828	0.08228	down
EPA (d5) (C20:5)	1.21	0.00826	1.50819	up*
Erucic acid (C22:1)	0.71	0.28367	0.73663	down
Adrenic acid (C22:4)	0.78	0.13189	0.98725	down
Docosapentaenoic acid (22:5)	0.32	0.02673	1.29346	down*
Docosahexaenoic acid (C22:6)	1.36	0.00630	1.56297	up*
Nervonic acid (C24:1)	0.03	0.00001	2.03213	down*

*Represents significantly changed metabolites (*P* < 0.05 and VIP > 1 were set as the threshold for significantly changed metabolites)

^a^*P* - value was calculated from Student's *t*-test

^b^VIP value, variable influence on projection (VIP) value was obtained from the OPLS-DA

^c^Up and down indicate an upward and downward trend in metabolites under cold acclimation

In saturated fatty acids, there was a significant decrease (*P* < 0.05) in tridecanoic acid while nonadecanoic acid was significantly increased (*P* < 0.05) compared with control group (Table 4). However, we found that there was no significant difference on other saturated fatty acids, including heptanoic acid, pelargonic acid, myristic acid, pentadecanoic acid, palmitic acid, arachidic acid, behenic acid, undecanoic acid, dodecanoic acid, and stearic acid. These results indicate that the extension pathways of fatty acids may be accelerated under cold acclimation.

**Table 4:** Differential expression of saturated fatty acids in serum of *S. prenanti* under cold acclimation (control vs. cold)

Metabolites name	Fold change	*P* - value^a^	VIP value^b^	Trend^c^
Heptanoic acid (C7:0)	1.05	0.60673	0.32995	up
Pelargonic acid (C9:0)	1.41	0.35900	0.56543	up
Undecanoic acid (C11:0)	0.78	0.18664	0.85945	down
Dodecanoic acid (C12:0)	0.92	0.09547	1.09965	down
Tridecanoic acid (C13:0)	0.3	0.00035	1.99140	down*
Myristic acid (C14:0)	1.64	0.08763	1.08984	up
Pentadecanoic acid (C15:0)	1.67	0.06011	1.11373	up
Palmitic acid (C16:0)	1.01	0.95521	0.02419	up
Stearic acid (C18:0)	0.74	0.23804	0.76315	down
Nonadecanoic acid (C19:0)	1.23	0.00430	1.58490	up*
Arachidic acid (C20:0)	1.41	0.37364	0.55927	up
Behenic acid (C22:0)	1.01	0.80675	0.20833	up

*Represents significantly changed metabolites (*P* < 0.05 and VIP > 1 were set as the threshold for significantly changed metabolites)

^a^*P* - value was calculated from Student's *t*-test

^b^VIP value, variable influence on projection (VIP) value was obtained from the OPLS-DA

^c^Up and down indicate an upward and downward trend in metabolites under cold acclimation

### ABC transporters and steroid degradation pathways are induced in response to cold acclimation

We found that ABC transporters were significantly enriched under cold acclimation (Fig. 4C). Further analysis revealed that D-xylose, 2’-Deoxyadenosine, and L-leucine were significantly increased (*P* < 0.05) under cold acclimation (Table 5). On the contrary, the levels of uridine and cytidine were significantly decreased (*P* < 0.05) under cold acclimation (Table 5). In steroid degradation, we found that the level of cholesterone was significantly increased (*P* < 0.05) while the level of epiandrosterone was significantly decreased (*P* < 0.05) under cold acclimation. These results indicate that ABC transporters and steroid degradation pathways is induced under cold acclimation.

**Table 5:** Differential metabolites of ABC transporters and steroid degradation pathways in serum of *S. prenanti* under cold acclimation (control vs. cold)

Metabolites name	Fold change	*P* - value^a^	VIP value^b^	Trend^c^
D-xylose	1.25	0.01716	1.50724	up*
Uridine	0.41	0.00477	1.70189	down*
Cytidine	0.44	0.01735	1.57697	down*
2’-Deoxyadenosine	1.17	0.03091	1.30656	up*
L-leucine	1.16	0.01829	1.51087	up*
Cholestenone	2.47	0.01331	1.53096	up*
Epiandrosterone	0.39	0.00300	1.62451	down*

*Indicating significantly changed in metabolites (*P* < 0.05 and VIP > 1 were set as the threshold for significantly changed metabolites)

^a^*P* - value was calculated from Student's *t*-test

^b^VIP value, variable influence on projection (VIP) value was obtained from the OPLS-DA

^c^Up and down indicate an upward and downward trend in metabolites under cold acclimation

### Purine metabolism and TCA cycle were inhibited under cold acclimation

As shown in Fig. 4C, we found that the purine metabolism was significantly (*P* < 0.05) enriched under cold acclimation. Further analysis showed that the levels of hypoxantine and uric acid were significantly (*P* < 0.05) decreased in the cold group, indicating that the purine metabolism pathway may be inhibit under cold acclimation. In addition, the TCA cycle pathway was also significantly (*P* < 0.05) enriched under cold acclimation. We found that the levels of L-malic acid and cis-aconitic acid were significantly (*P* < 0.05) decreased compared with control group, indicating that the TCA cycle may be inhibited under cold acclimation.

## Discussion

In the present study, we cultured *S. prenanti* in two environment temperatures for 30 days, and analyzed the effects of low temperature on the cold-water fish using a series of physiological and biochemical indicators, and LC-MS metabolomics. We found that fish exhibited different metabolomic characteristics under cold acclimation, while the immune status was not affected under cold acclimation. Glucose is the preferred energy source for consumption in the early stages of cold stress in fish [24, 25]. According to previous studies, the level of serum glucose in yellow croaker [26] and goby [27] is decreased under cold stress. In the present study, we found that the serum glucose in *S. prenanti* was significantly decreased under cold acclimation, indicating that the decrease in glucose may be due to the use of glucose as the main energy by cold-water fish under cold acclimation. In addition, metabolic analysis showed that cold treatment increased the level of glyceric acid, indicating that glycerolipid metabolism is induced under cold acclimation.

The results of warm-water fish under acute cold stress conditions and long-term cold exposure are different, which may be related to the duration of cold exposure. In large yellow croaker, the level of triglyceride in the plasma increases under 9℃ for 6 h [26]. In tilapia, the triglyceride level significantly increases under 9℃ for 2 h and 6 h, and returns to the normal level after 12 h treatment [15]. However, in silver catfish, it was found that the level of triglyceride significantly decreases during 21 days of exposure to 15℃ [25]. In this study, the level of triglyceride was significantly increased under cold acclimation. We speculate that this may be the difference between cold-water fish and warm-water fish. These results indicate that cold acclimation increases the level of serum triglyceride, which may provide the fuel for *S. prenanti* to cope with low temperature.

According to previous reports on fish, total cholesterol (TC) is an important energy substance under cold stress [28]. In yellow catfish, LDL-C and TC significantly increases under low temperature stress [29]. These results are consistent with our study. We speculate that fish may need to increase LDL-C and TC in serum to provide more energy to support their metabolic needs under cold conditions. It is reported that in humans, elevated levels of LDL-C and TC may be a potential risk factor for cognitive impairment [30] and a potential indicator of dyslipidemia in diabetes [31]. However, we speculate that the high levels of these lipid substances may be a normal adaptive physiological response in cold-water fish to meet their energy needs under long-term cold acclimation environment. In addition, cholestenone was significantly increased in the cold group, and cholestenone is an intermediate oxidation product of cholesterol [32], indicating that fish promotes the degradation of steroids under cold acclimation. The oxidation of cholesterol to cholestenone releases energy, which may be used for cellular metabolic activities, ATP synthesis, and thermogenesis.

The intestinal mucosa can regulate the activity of immune cells against pathogenic microorganisms and exercise immune defense functions by secreting chemokines and cytokines from intestinal epithelial cells [33]. Most pro-inflammatory cytokines include IL-1β, IL-8, TNF-α, can lead to an increase in intestinal mucosal permeability, while some anti-inflammatory cytokines including IL-10 and TNF-β inhibits the deterioration of intestinal inflammation [34]. The interaction between pro-inflammatory and anti-inflammatory factors maintains immune balance [35]. In this study, there were no significant changes in anti-inflammatory and pro-inflammatory factors in the serum and intestine of *S. prenanti* under cold acclimation. These results indicate that cold acclimation has no effects on the immune status of *S. prenanti* and may not cause intestine inflammation in *S. prenanti*. This may be an important reason why *S. prenanti* maintain normal growth and development without being affected by cold acclimation.

In addition to amino acids as components of proteins and peptides, some amino acids also regulate the key metabolic pathways necessary for maintaining osmotic pressure, growth, reproduction, and immunity [36]. Previous studies have been shown that cold stress significantly increases the level of L-asparagine in yellow catfish [29]. In mice, asparagine elevates rectal temperature and promotes thermogenesis in brown adipose [37]. In the present study, we also found that the level of L-asparagine was significantly increased under cold exposure. In addition, we found that the levels of L-leucine and ketolecine were upregulated under cold acclimation, while S-adenosylmethionine and L-methionine were downregulated under cold acclimation. According to previous studies, L-leucine has beneficial effects on the intestinal morphology and immune function of piglets [38]. In addition to being a precursor of L-glutamine [36], L-leucine can also increase protein synthesis and reduce protein hydrolysis by activating the mTOR signaling pathway in piglets and human intestine [39]. It has been reported that increasing protein synthesis can improve the level of muscle protein in mice to promote muscle growth [40], which may be the reason why *S. prenanti*, as a cold-water fish, can maintain normal growth and development under cold acclimation.

In addition, Omega-3 unsaturated fat acids have been proved to prevent intestinal dysfunction and immunity induced by cold exposure [41]. The reduction of unsaturated fatty acids may be an important reason for the decline in fish growth [42]. In the present study, we found the levels of DHA and EPA were significantly increased under cold acclimation, indicating that DHA and EPA are important to maintain normal growth and development under low temperature. As a long chain monounsaturated fatty acid, nervonic acid is a key component of sphingolipids, which participates in many biological processes, such as cell membrane formation, apoptosis, and neurotransmission [43, 44]. The decrease of nervonic acid may reflect its important roles in the cold acclimation response in *S. prenanti*. In nile tilapia, the level of unsaturated fatty acid is increased after cold stress [45], which is consistent with our results. We speculate that *S. prenanti* may adapt to long-term low temperature by accelerating the metabolism of unsaturated fatty acids.

ABC transporters include various types such as oligosaccharide, polymer and lipid transporters, monosaccharide transporters, and phosphate and amino acid transporters. ABC transporters can catalyze the hydrolysis of ATP and utilize the energy released in this reaction to perform molecular transport [46]. In the present study, we found that uridine was significantly decreased in serum under cold acclimation. Previous study has demonstrated that cells can utilize uridine as a source of nutrition and energy when glucose is limited [47]. In addition, our results showed that cytidine was significantly decreased, which is consistent with the results of the yellow catfish serum [29]. The results indicate that *S. prenanti* may provide energy by accelerating the transport of uridine and cytidine under cold acclimation. In the liver of yellow drum, ABC transporters is induced under cold stress [48]. The changes in ABC transporters may reflect their important roles in the cold acclimation response in *S. prenanti*.

Uric acid and hypoxanthine are products of purine metabolism, and the decrease in their levels may reflect the inhibition of purine metabolism pathway. Purine metabolism is closely related to redox balance. Reactive oxygen species (ROS) can be produced during the oxidation of hypoxanthine [49]. The decrease of uric acid and hypoxanthine levels may reflect the regulation of redox balance in fish cells under low temperature environment. Reducing the levels of uric acid and hypoxanthine may help reduce oxidative stress and free radical production, and protect cells from damage. In contrast, the results of sea bream liver [50] and yellow catfish serum [29] showed that purine metabolism was induced, and the levels of uric acid and hypoxanthine are up-regulated under cold environments. This may be the difference between cold-water fish *S. prenanti* and other fish in adapting to cold. TCA cycle performs the basic function of oxidizing nutrients to support cell bioenergetics [51], and energy supply [52]. Our results showed that L-malic acid and cis-aconitic acid, which are intermediate metabolites of TCA cycle, are significantly down-regulated in the cold group, indicating that TCA cycle is inhibited under cold acclimation, which is consistent with the results of black rockfish serum [53].

## Conclusion

The present study demonstrated that *S. prenanti* exhibited different metabolic changes in serum under cold acclimation to maintain biological functions. In addition, metabolomics analysis results revealed that cold acclimation altered expression of metabolites involved in amino acid, the unsaturated fatty acids metabolism, purine metabolism, TCA cycle. The above results provide a new insight into the physiological responses of cold-water fish to adapt to cold acclimation.

## Materials and methods

### Experimental animal

Fish were maintained for cultivation at the Fish Breeding Center of Sichuan Agriculture University (Ya’an, China), with an average body weight of 128.17 ± 2.99 g (*n* = 120). In October 2022, a total of 120 fish were temporarily raised in a water tank (1130 L) at a temperature of around 21℃ for acclimation. During the acclimation period and subsequent experiments, fish were fed 1.0% body weight twice a day with commercial diet (G68, Sichuan, China) (8:30 am and 5:30 pm). We replaced the water with aerated water twice a day and maintained sufficient oxygen supply (dissolved oxygen > 8.0 mg/L) in the tank. The fish were maintained on a 12 h: 12 h light/dark cycle. After temporarily raising for a month, 90 fish were randomly selected from 120 fish in the water tank (1130 L) and transferred to 6 small water tanks (160 L), with 15 fish in each tank. We used 3 small water tanks as the control group (125.00 ± 2.67 g) and the other 3 small water tanks as the cold group (124.87 ± 3.44 g). Before sampling, we randomly select 5 fish from each tank, with 15 fish from each group. 8 fish were allocated for serum biochemical analysis, and 7 fish were designated for serum metabolomics analysis and mRNA quantification analyses of intestine samples.

A chiller was used for chronic cooling, with a cooling rate of 1℃/day to 6℃. After stabilizing the temperature, samples were taken from the control and cold groups after 30 days of acclimation, and fasting was performed for 24 h before sampling.

### Serum collection

The fish were anesthetized with 0.02% tricaine methanesulfonate (MS-222, J&K Scientific, Beijing, China). Then, we disinfected the fish body with 75% ethanol, and then collected blood from the tail vein. Blood was collected in a tube and stood at room temperature for 1 h for coagulation and stratification. Then, blood was centrifuged at a speed of 4000 rpm at 4℃ for 10 min and supernatant was retained and frozen at -80℃ until further analysis.

### Biochemical analysis

From 8 fish each group (*n* = 8), serum total cholesterol (TC), glucose (GLU), triglycerides (TG), high density lipoprotein (HDL-C), and low density lipoprotein (LDL-C) were determined with the help of commercial kits (NJBI Clinical Reagent, Nanjing, China) by Hitachi 7020 Automated Biochem (Hitachi, Tokyo, Japan). Serum tumor necrosis factor-β (TNF-β) and interleukin-1β (IL-1β) were measured using commercially available ELISA kits (Jiangsu Meimian Industrial Co., Ltd., Nanjing, China).

### qPCR analysis

From 7 fish each group (*n* = 7), hindgut (the last section of the intestine) was squeezed and removed with forceps to exclude the intestinal content. Samples were stored at -80 °C before RNA extraction. Total RNA was extracted from *S. prenanti* hindgut with Trizol reagent (TaKaRa, Dalian, China). The concentration of total RNA was measured at 260 nm by Nano Drop 2000 (Termo, MA, USA). RNA integrity (Table S3) was assessed by the Agilent Bioanalyzer 2100 system (Agilent, CA, USA). A total amount of 1 µg RNA was used to synthesize cDNA. The first strand of cDNA was synthesized using the Prime Script RT Reagent kit (Takara, Tokyo, Japan). The primer sequences used for qPCR are shown in Table S1. The qPCR reactions were utilized to quantify the expression of genes with CFX96TM Real-Time PCR Detection System (Bio-Rad, Hercules, USA). The reaction cycling conditions were 95 °C for 30 s, followed by 40 cycles at 95 °C for 10 s and 60 °C for 30 s. Each reaction (in 10 µL) was performed with 5 µL ChamQ SYBR qPCR Master mix (Vazyme, Nanjing, China), 0.4 µL for primers, 3.4 µL of sterilized double distilled water and 0.8 µL cDNA template. Expression levels were normalized to the expression of the reference gene β-actin by the 2^−ΔΔCt^ method.

### Metabolomics analysis by LC-MS

From 7 fish each group (*n* = 7), we performed liquid chromatography gradient separation on the processed serum samples. Chromatography was carried out with an ACQUITY UPLC ® HSS T3 (150 × 2.1 mm, 1.8 μm) (Waters, Milford, MA, USA). The temperature of the chromatographic column was maintained at 40℃. The flow rate and injection volume were set to 0.25 mL/min and 2 mL/min, respectively. Briefly, the mobile phase used for LC-ESI (+) - MS analysis consisted of a solution of (B1) 0.1% formic acid in acetonitrile (v/v) and (A1) 0.1% formic acid aqueous solution (v/v). Separation was conducted under the following gradient: the gradient remained at 2% B1 for the first 1 min, and linearly increased to 50% in the next 8 min. From 9 min to 12 min, it gradually increased to 98% B1 and remained at this level for the next 1.5 min. Within 13.5–14 min, the gradient immediately decreased from 98% B1 to 2% B1 and remained as such until 20 min. LC-ESI (-) - MS analysis was carried out with (B2) acetonitrile and (A2) ammonium formate (5mM). The gradient separation program was the same as LC-ESI (+) - MS analysis, the program gradient was from 0 to 14 min, except for replacing B1 with B2 and maintaining 2% B2 for only 3 min after 14 min [54].

Mass spectrometric detection of metabolites was performed on Q Exactive (Thermo Fisher Scientific, USA) with ESI ion source. We measured metabolites using both positive and negative ion modes with 3.50 kV and − 2.50 kV of spray voltage for ESI (+) and ESI (-), respectively [55].

### Data analysis

The data was analyzed using GraphPad Prism 9 analysis software and shown as mean ± SEM. We compared the differences between the control and cold groups using two-tailed unpaired Student’s *t*-test at the 0.05 significance level (*P* < 0.05). Convert the original mass spectrometry offline file to mzXML file format using the MSConvert tool in the ProteoWizard software package. The XCMS software package was used for peak detection, filtering, and alignment processing.

Principal component analysis (PCA) showed the distribution of origin data and supervised orthogonal partial leastsquares-discriminant analysis (OPLS-DA) was used to obtain the level of group separation. We calculated the variable projection importance (VIP) values using OPLS-DA dimensionality reduction method, and calculated the inter-group difference. When the *P* < 0.05 and the VIP > 1, it was considered that the metabolites have statistical significance. The Kyoto Encyclopedia of Genes and Genomes (http://www.genome.jp/kegg/) was used to search for the pathways of the metabolites.

### Supplementary Information

Supplementary Material 1.

Supplementary Material 2.

Supplementary Material 3.

## Data Availability

All data generated in this study are included in the main article and its supplementary files. The data reported in this paper have been deposited in the OMIX, China National Center for Bioinformation / Beijing Institute of Genomics, Chinese Academy of Sciences (https://ngdc.cncb.ac.cn/omix: accession no.OMIX005898).

## Abbreviations

S. prenanti: Schizothorax prenanti
GLU: Glucose
TC: Total cholesterol
TG: Triglycerides
HDL-C: High density lipoprotein
LDL-C: Low density lipoprotein
IL-8: Interleukin-8
IL-1β: Interleukin-1β
IL-10: Interleukin-10
TNF-α: Tumor necrosis factor-α
TNF-β: Tumor necrosis factor-β
EPA: Eicosapentaenoic acid
DHA: Docosahexenoic acid
DPA: Docosapentaenoic acid
PCA: Principal component analysis
OPLS-DA: Orthogonal partial leastsquares-discriminant analysis
VIP: Variable projection importance
