# Supplementary material for: Effects of cold acclimation on serum biochemical parameters and metabolite profiles in Schizothorax prenanti
Source: BMC Genomics. 2024 Jun 1;25:547. doi: 10.1186/s12864-024-10483-z (PMC11143564; doi:10.1186/s12864-024-10483-z)
Supplement: Supplementary file 1 — Supplementary Material 1. [file 12864_2024_10483_MOESM1_ESM.docx]

Table S1 The sequences of primers used in this study.

| Primer name | Primer sequence (5′–3′) | Tm (℃) | Size (bp) |
| --- | --- | --- | --- |
| TNFα-F  TNFα-R | CACCAAAACCACCACAGAACTC  TAAAGCAAACACTCCAAAAAAG | 56.0 | 76 |
| IL-8-F  IL-8-R | TGTGAAGTTGAGCCTGAAGATA  AGTAGGACTGTGCCCATCGGTA | 56.0 | 76 |
| IL-1β-F  IL-1β-R | GGTGGTGAACATCATCATTGC  AGACGCTCTTCGATCACATTC | 54.0 | 120 |
| IL-10-F  IL-10-R | TTCGTTCAGTAATGGTTCCAAGTCA  TGTGGAGGGCTTTCCTGTGAG | 60.4 | 103 |
| TNF-β-F  TNF-β-R | CTGGGCTGGAAGTGGATA  AGTAAAAGATGGGCAGTGGGT | 56.0 | 190 |
| β-actin-F  β-actin-R | TTCTTGGGTATGGAGTCTTG  AGGTCCTTACGGATGTCG | 59.0 | 83 |

F: forward primer; R: reverse primer.
