# Supplementary material for: Effects of cold acclimation on serum biochemical parameters and metabolite profiles in Schizothorax prenanti
Source: BMC Genomics. 2024 Jun 1;25:547. doi: 10.1186/s12864-024-10483-z (PMC11143564; doi:10.1186/s12864-024-10483-z)
Supplement: Supplementary file 3 — Supplementary Material 3. [file 12864_2024_10483_MOESM3_ESM.docx]

Table S3. Results of sample RNA quality determination.

| Sample name | RIN-value | 28S/18S |
| --- | --- | --- |
| Con-1 | 8.6 | 2.19 |
| Con-2 | 8.3 | 2.06 |
| Con-3 | 8.3 | 2.09 |
| Con-4 | 8.9 | 2.32 |
| Con-5 | 8.6 | 2.13 |
| Con-6 | 8.5 | 2.2 |
| Con-7 | 8.1 | 2.07 |
| Cold-1 | 8.9 | 2.29 |
| Cold-2 | 8.6 | 2.24 |
| Cold-3 | 8.3 | 2.18 |
| Cold-4 | 8.7 | 2.10 |
| Cold-5 | 9.1 | 2.62 |
| Cold-6 | 8.5 | 2.29 |
| Cold-7 | 8.9 | 2.46 |
